# Maternal Ferrous Sucrose Supplementation Improves Reproductive Performance of Sows and Hepatic Iron Stores of Neonatal Piglets

**DOI:** 10.3390/ani15030343

**Published:** 2025-01-25

**Authors:** Wen Tian, Xiaofan Ma, Hongwei Liu, Zhefeng Wang, Chunxue Liu, Chunyan Xie

**Affiliations:** 1 Key Laboratory of Agro-ecological Processes in Subtropical Region, Institute of Subtropical Agriculture, Chinese Academy of Sciences, Changsha 410125, China; tianw2022@isa.ac.cn (W.T.);; 2 Anyou Biotechnology Group Co., Ltd., Taicang 215437, China; 3 Tianjin Key Laboratory of Animal Molecular Breeding and Biotechnology, Tianjin Livestock and Poultry Health Breeding Technology Engineering Center, Institute of Animal Science and Veterinary, Tianjin Academy of Agricultural Sciences, Tianjin 300381, China

**Keywords:** pregnant sows, iron supplement, serum iron, growth, offspring

## Abstract

As one of the most important trace elements required by sows, especially in the late gestation period, iron plays a crucial role in the growth and development of a fetus. To explore the effects of dietary supplementation with ferrous sucrose on the reproductive performance of sows and the hepatic iron stores in offspring, sixty primiparous Landrace × Yorkshire sows on day 95 of gestation with an average body weight of 174.1 ± 7.7 kg were randomly assigned to two groups of a basic diet (control) and a basic diet supplemented with 109 mg/kg ferrous sucrose (FS) in a fully randomized block design. The trial lasted for 20 days. The results showed that maternal supplementation with ferrous sucrose significantly increased litter weight (*p* = 0.002) in neonatal piglets. Compared with the control group, the serum iron and serum transferrin saturation of farrowing sows increased by 45.67% (*p* = 0.002) and 37.01% (*p* = 0.033), respectively, and umbilical cord serum iron (*p* = 0.012) also increased in the FS group. Finally, the serum iron (*p* < 0.001) and hepatic iron stores (*p* = 0.071) of neonatal piglets were both increased to varying degrees. Taken together, supplementation of pregnant sows with ferrous sucrose has positive effects on the growth and hepatic iron stores of their offspring.

## Simple Summary

Iron supplementation is necessary for pregnant sows, which is not only beneficial for the generation of red blood cells in sows, but also essential for fetal growth. This study explored the effects of supplementation of pregnant sows with ferrous sucrose in the late gestation period on reproductive performance and iron stores in piglets. The results indicated that maternal supplementation with ferrous sucrose increased litter weight and average weight and promoted the development of small intestinal villi. It also increased hepatic iron stores in neonatal piglets. This study provides new ideas and solutions for preventing iron deficiency in neonatal piglets.

## 1. Introduction

Iron is an essential micronutrient for almost all living organisms and participates in various metabolic processes [1]. The most famous function of iron is its presence in hemoglobin (Hb), which participates in the transport of oxygen. However, iron plays a [2] crucial role in cellular processes such as DNA synthesis, epigenetic regulation, and cellular respiration in humans and mice [2,3,4], especially during maternal pregnancy. In the last third of pregnancy, maternal and fetal iron demands increase exponentially in swine [5]. Most of the iron absorbed and stored by the sows during pregnancy is supplied to improve maternal red blood cell (RBC) mass and meet the needs of placental and fetal growth [6]. However, during pregnancy, low iron stores in the fetus directly affect the growth rate and immunity of piglets in the later stages [7] and even increase mortality rates [8]. Due to the prolificacy of sows, insufficient iron stores in full-term neonatal piglets are attributed to the inability of pregnant sows to provide sufficient iron for each fetus [9,10]. On the contrary, the red blood cell and iron status (including liver iron content) of neonatal piglets are mainly not determined by litter size [11]. However, the main purpose of supplementing iron in pregnant sows is to increase their iron levels, and then to provide additional iron for their fetuses to meet the erythropoietic needs [9,12]. The potential reason is that increasing the mother’s iron status will strengthen the process of iron transfer from the mother to the fetus through the placenta, thereby increasing the iron content in the liver of offspring [10].

The NRC [13] recommends that the dietary iron requirement of gestating sows is 80 mg/kg feed. However, even with the addition of phytase, the apparent total tract digestibility of iron in a corn–soybean meal diet fortified with ferrous sulfate was only about 25% [14,15]. Therefore, some studies attempted to increase maternal iron status and fetal hepatic iron stores by treating pregnant sows with various forms of iron supplements [16,17], often exceeding the requirements [18]. Bhattarai et al. found that intramuscular injection of two doses of 12.5 mL dextran iron (equivalent to 2500 mg iron) 2 weeks apart at mid-gestation resulted in no changes in sows’ hematological variables or piglets’ hematological indicators compared to the saline injection group when 83 mg/kg iron supplements were added to the diet [16]. However, when different levels of ferrous glycine chelate or ferrous sulfate (50–140 mg Fe/kg diet) were supplemented to sows from 85 days of pregnancy to delivery, sows supplemented with 110 mg/kg ferrous glycine chelate showed better Hb concentration, tissue iron content, and blood biochemical indicators in their newborn piglets compared to other forms of supplementation [18]. Previous studies have shown that sows can benefit from an exogenous iron supplementation dose of approximately 140 mg/kg. Buffler et al. confirmed that supplementing 142 mg/kg ferrous sulfate in the basal diet of sows from fertilization to delivery significantly improved the litter size and weight of neonatal piglets [19]. Additionally, an analysis of the iron content in commercial diets of gestating sows from 391 farms surveyed revealed that the average iron supplementation level was 3.93 times that of the NRC requirement and 3.37 times that of China’s Feeding Standard [20]. However, supplementing iron supplements to pregnant sows has not fully achieved the effect of improving the iron status of sows and neonatal piglets. It may be related to the treatments at different stages of pregnancy, or using different types or doses of iron supplements, or in different ways it is administered in sows [16,21].

From inorganic iron salts (represented by ferrous sulfate) to chelated forms of iron (mainly composed of amino acid chelated iron, fumarate chelated iron, and lactate chelated iron), and then to lactoferrin, iron supplements have been continuously improved and innovated. Although ferrous sulfate is inexpensive, it has low bioavailability and can induce gastrointestinal complications [22]. The chelated forms of iron and lactoferrin are recognized for their higher absorption rate and better stability than inorganic iron [23], but they are less commonly used in pig farms due to their high price [9]. Therefore, researchers are committed to developing a new type of iron supplement with good stability, low price, and easy absorption. Sucrose has a wide range of sources and can increase the absorption of metal ions by contributing hydroxyl electrons to bind with them. It is an excellent and stable metal ion chelating agent [24]. Ferrous sucrose (FS) is a newly developed natural sugar–iron complex, which is a complex combining the glycoside oxygen of sucrose with iron (Ⅱ) through a covalent bond. In previous evaluations of the effectiveness and tolerance of FS, it was found that supplementing piglets with 60 mg/kg (calculated as iron) of FS in their diet significantly increased their average daily weight gain and serum iron levels compared to the control group. In a tolerance evaluation, it was found that when the amount of FS added to the diet of weaned piglets reached 900 mg/kg (calculated as iron), the blood biochemical indicators, organ indices, and organ tissue pathology of piglets were within the normal range, and there was no significant difference from the effective dose (the above data have not been publicly released). Therefore, this study hypothesized that supplementing FS in the basal diet of late pregnant sows to achieve 140 mg/kg iron can improve the reproductive performance of sows and the hepatic iron stores of neonatal piglets.

## 2. Materials and Methods

### 2.1. Ethics Statement

The experimental procedures used in this study were approved by the Protocol Management and Review Committee of the Institute of Subtropical Agriculture of the Chinese Academy of Science. Pregnant sows were raised and piglets were killed in compliance with protocol ISA-2021-0069 from the Institute of Subtropical Agriculture on Animal Care in Changsha, China.

### 2.2. Ferrous Sucrose Samples

FS was produced by Nanning Zeweier Feed Co., Ltd. (Guangxi, China), with an iron content of 9.8%.

### 2.3. Animals, Diets, and Experimental Design

The trial was conducted at the Pig Production Base of Anyou Group (Jintan, Changzhou, Jiangsu). A total of sixty healthy primiparous Landrace × Yorkshire (LY) sows (mated with the same boar) on the 95th day of gestation with an average body weight of 174.1 ± 7.7 kg were used in this study. The sows were in gestation cages during gestation and transferred to farrowing cages on the 110th day of gestation. Throughout the whole trial, the pregnant sows were raised in individual cages with free access to water. According to the nutrient concentration of the feed and body condition score, the sows were provided 3 kg diet per day and fed twice a day.

The sows were randomly assigned to two groups of a basic diet (control) and a basic diet supplemented with 109 mg/kg FS (supplied 10.7 mg/kg of iron in the diet, FS) in a fully randomized block design. There were thirty sows in each group, and one sow was one experimental unit. The trial lasted for 20 days, from the 95th day of pregnancy to parturition. Other operations were carried out in accordance with the management of commercial farms.

The basic diet was formulated to meet or exceed all nutrient requirements for a sow in late pregnancy according to the NRC [13] (Table 1).

### 2.4. Recording and Sample Collection

On the 95th day of pregnancy and the day of delivery, blood was harvested from the ear vein of sows by venipuncture for measurement of hemoglobin and hematocrit. The number of total piglets, alive piglets, stillborn piglets, and intrauterine growth retardation (IUGR) piglets and the litter weight of piglets were recorded at delivery. IUGR refers to the impaired growth and development of mammalian embryos or a fetus or its organs during pregnancy [25]. IUGR piglets refer to piglets whose birth weight is 1.5 SD lower than the average litter weight [26]. At the same time, 8 sows with reproductive performance close to the group average in the same delivery house were selected from each group for sample collection. The blood samples from the ear vein of sows and umbilical cords were collected [26], and serum samples were separated (1800 g, 10 min) [27] and stored at −80 °C for subsequent mineral element analysis. Placental tissue samples were collected after parturition and immediately frozen in liquid nitrogen and then stored at −80 °C for further analyses [26]. Blood and tissue samples from piglets sourced from the litter of selected sow were obtained within 24 h of birth. Neonatal piglets were euthanized by intravenous injection of 0.5 mL/kg body weight of sodium pentobarbital solution for collecting blood samples from the jugular vein and liver tissue samples [10]. Serum samples were collected after centrifugation. All samples of piglets were stored at −80 °C for subsequent analysis.

### 2.5. Analytical Methods

#### 2.5.1. Determination of Hemoglobin and Serum Iron Relative Indices

Hemoglobin and hematocrit were determined according to the operating instructions using an automatic hemoglobin analyzer (ACON Biotechnology Co., Ltd., Hangzhou, China) [28]. Serum iron (SI) and unsaturated iron binding capacity (UIBC) were assayed by commercial kits (IRON Gen.2, 200 tests and UIBC, 100 tests, Roche) with a fully automatic biochemical analyzer (Cobas c311, Roche Diagnostics USA, Rotkreuz, Switzerland) [29].

Transferrin saturation (TS) was calculated as

TS = SI/(SI + UIBC) × 100%

where SI and UIBC were obtained from a blood biochemical analysis.

#### 2.5.2. Measurement of Mineral Element Contents in Serum and Tissue Samples of Liver and Placenta

An amount of 300 μL of serum was added to 5 mL of pure nitric acid and 1 mL of 30% hydrogen peroxide and subsequently digested in a microwave digestion instrument (ETHOS UP, Milestone, Italy) according to the procedures described by Liu et al. (2024) [28]. After complete digestion, the digestion tube with digested liquid was placed in a Rush acid meter (VB24UP, LABTECH, Beijing, China) at 180 °C to remove excess nitric acid and then diluted to 10 mL with 1% nitric acid. Finally, calcium (Ca), phosphorus (P), magnesium (Mg), copper (Cu), iron (Fe), manganese (Mn), and zinc (Zn) in the dilution solution were detected using an inductively coupled plasma atomic emission spectrometer (5110 ICP-OES, Agilent Technologies, Santa Clara, CA, USA) [17]. Three parallel tests were set up for each sample, and all results were calibrated using mineral element standards and validated by the accuracy of analytical methods. The accuracy is based on the relative standard deviation of metal ion concentration in the same sample digestion solution being less than 15%.

Liver samples and placenta samples were freeze-dried by a Refrigerant-drier (Scientz-100F, SCIENTZ, Ningbo, China) and ground into powder. According to the ratio of 0.1–0.2 g liver sample to 8 mL pure nitric acid and 1 mL 30% hydrogen peroxide, the procedure of digestion and subsequent steps is the same as those for the blood samples [17].

#### 2.5.3. Histological Examination

After slaughtering, the duodenum, jejunum, and ileum samples (each about 2 cm) of piglets were collected and immediately placed in 4% paraformaldehyde and embedded with paraffin before they were cut into 5 µm slices. The tissue sections were stained with hematoxylin and eosin (H&E) after being dewaxed and then were measured using a microscope [30,31].

#### 2.5.4. Real-Time Quantitative RT-PCR

Total RNA was extracted from placental tissue using Trizol reagent (Invitrogen, Carlsbad, CA, USA), as previously described by Xie [32], and treated with RNase-free DNase for removing DNA contamination and then reverse transcribed to cDNA using a Transcriptor First Strand cDNA Synthesis Kit (Takara Biomedical Technology (Beijing) Co., Ltd., Beijing, China) according to the manufacturer’s protocol. Primer sequences of the relevant genes for the real-time quantitative polymerase chain reaction (RT-qPCR) were designed by Prime 5.0 and synthesized by Sangon Bioengineering (Shanghai, China) Co., Ltd., as shown in Table 2. The RT-qPCR analysis was performed in a Roche fluorescence quantitative PCR instrument (Light Cycler 480II, Basel, Switzerland) according to the handbook of the kit (Accurate Biology, Changsha, China). The relative expression level of genes related to iron transport was calculated using the 2^−ΔΔct^ method after normalization with β-actin as a housekeeping gene [33].

### 2.6. Statistical Analysis

Before the statistical analysis, all data were examined for normality using a normal distribution plot (UNIVARITE program). All statistical analyses were performed using the independent samples *t*-test of SPSS 16.0 (SPSS Inc., Chicago, IL, USA). For the analysis of reproductive performance and the hematological and serum iron indicators of sows, dietary treatment is considered a fixed effect, while litter and sows’ body weight are considered random factors. Figures were prepared using GraphPad Prism version 8.0; *p* ≤ 0.05, *p* ≤ 0.01, and *p* ≤ 0.001 were considered significant differences between the two groups, represented by asterisks *, **, and ***, respectively. The data are reported as means with standard error of the mean (SEM).

## 3. Results

### 3.1. Maternal Ferrous Sucrose Supplementation Had Little Effect on Blood Hemoglobin and Hematocrit in Sows

Before and after the study, the contents of hemoglobin and hematocrit of the sows were measured. It was found that regardless of before and after the study, the hemoglobin and hematocrit values of all sows involved in the study were similar. There was no difference in the blood hemoglobin and hematocrit of sows between the FS group and the control group during delivery (*p* > 0.05) (Table 3).

### 3.2. Maternal Ferrous Sucrose Supplementation Improved Serum Iron Level in Sows and Offspring

**Figure 1:**
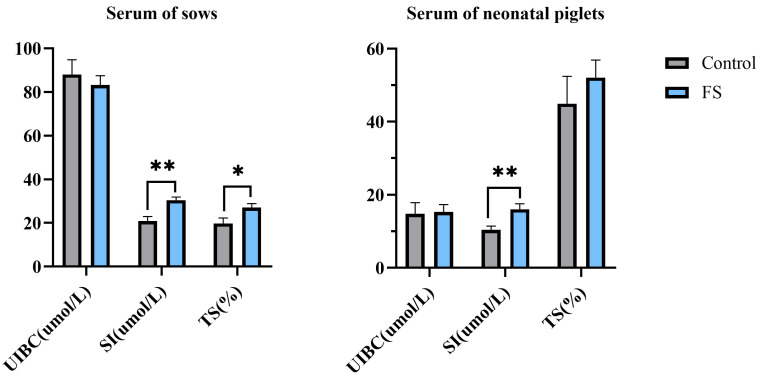
Effects of supplementation of pregnant sows with ferrous sucrose on serum UIBC, SI, and TS in sows and neonatal piglets. FS = ferrous sucrose, UIBC = unsaturated iron binding capacity, SI = serum iron, TS = transferrin saturation, TS = SI/(SI + UIBC) × 100%. Data are presented as mean with SEM. Statistical significances were set at * *p* ≤ 0.05 and ** *p* ≤ 0.01 by the independent *t*-test.

Supplementation with FS in the diet of sows during late pregnancy significantly increased SI levels and the TS of sows by 45.67% (*p* = 0.002) and 37.01% (*p* = 0.033), respectively. Supplementation with FS in the diet of sows during late pregnancy significantly increased the SI levels in neonatal piglets by 54.34% (*p* = 0.009) (Figure 1).

### 3.3. Maternal Ferrous Sucrose Supplementation Increased Litter Weight and Average Weight of Neonatal Piglets

The reproductive performance of sows is shown in Table 4. When pregnant sows were supplemented with FS, the numbers of piglets in the litter, alive piglets, IUGR piglets, and stillbirths remained unchanged compared with the non-supplemented control (*p* > 0.05). However, the piglets from sows receiving the FS supplementation treatment showed an increased birth litter weight of 20.74% (*p* = 0.002).

### 3.4. Maternal Ferrous Sucrose Supplementation Tended to Increase Duodenal Villus Height of Neonatal Piglets

**Figure 2:**
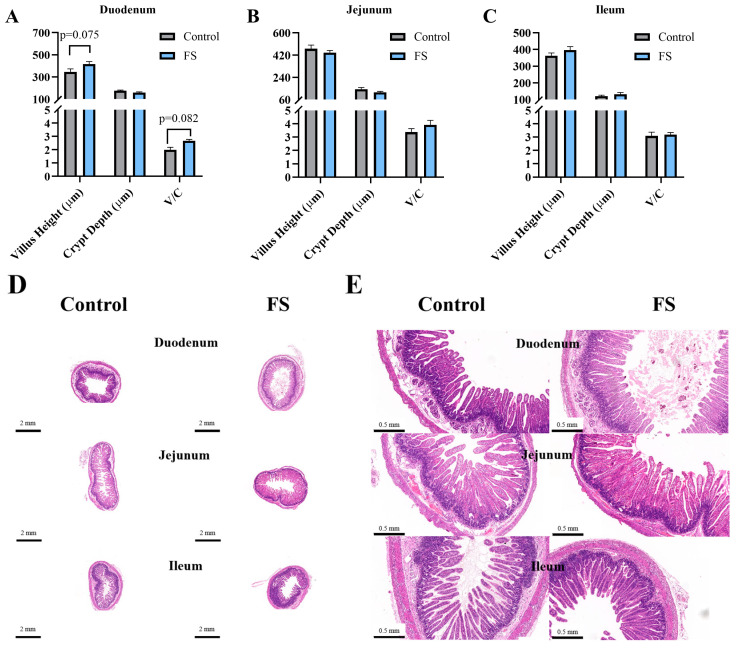
Effects of supplementation of pregnant sows with ferrous sucrose on intestinal morphology in neonatal piglets. FS = ferrous sucrose. (**A**–**C**) Villus height and crypt depth of the duodenum, jejunum, and ileum. (**D**) and (**E**) H&E staining of the duodenum, jejunum, and ileum at different magnifications. V/C = villus height/crypt depth, FS = ferrous sucrose. Data are presented as mean with SEM.

The morphology of the small intestine villi of neonatal piglets is depicted in Figure 2A–E. Supplementation with FS in the diet of sows during late pregnancy resulted in no changes in the small intestine morphology of neonatal piglets compared with the non-supplemented control (*p* > 0.05).

### 3.5. Maternal Ferrous Sucrose Supplementation Reduced Serum Calcium and Zinc in Sows

**Figure 3:**
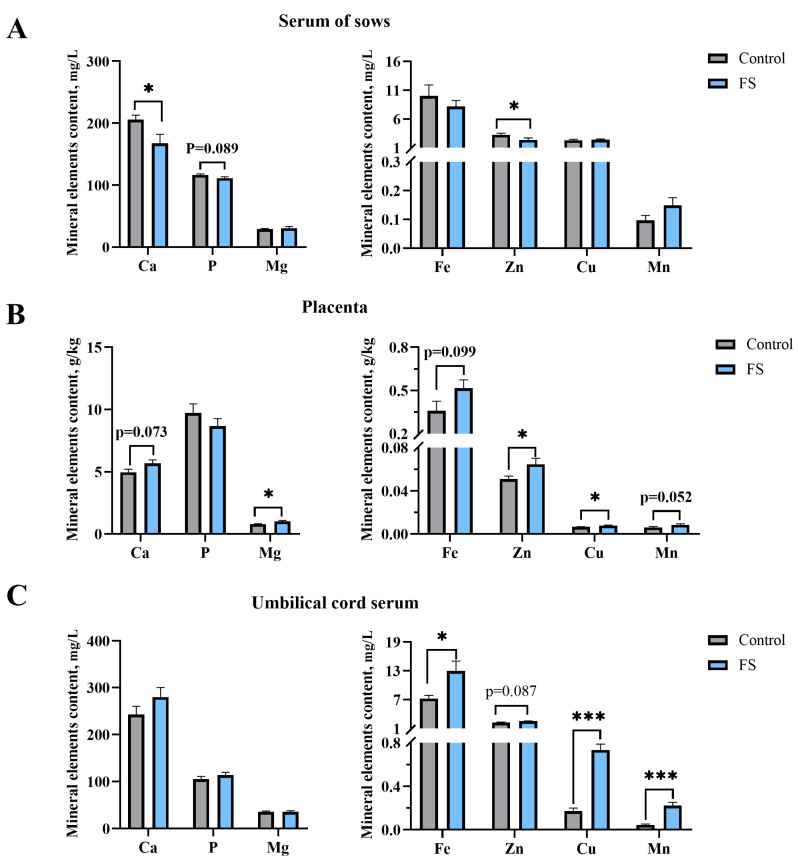
Effects of supplementation of pregnant sows with ferrous sucrose on the mineral element content in the serum of sows and in placenta and umbilical cord serum: (**A**) the mineral element content in the serum of sows; (**B**) the mineral element content in the placenta; (**C**) the mineral element content in the umbilical cord serum. FS = ferrous sucrose, Ca = calcium, P = phosphorus, Mg = magnesium, Fe = iron, Zn = zinc, Cu = copper, Mn = manganese. Data are presented as mean with SEM. Statistical significances were set at * *p* ≤ 0.05 and *** *p* ≤ 0.001 by the independent *t*-test.

The serum mineral element contents of the sows are shown as Figure 3. It is indicated that supplementation with FS in the diet of the sows during late pregnancy did not alter the content of non-heme iron (*p* > 0.05) but decreased the serum Ca and Zn contents of the sows by 18.65% (*p* = 0.030) and 28.13% (*p* = 0.050), respectively (Figure 3A).

### 3.6. Maternal Ferrous Sucrose Supplementation Enhanced the Contents of Trace Elements in the Placenta and Umbilical Cord Serum

As shown in Figure 3, FS supplementation of the sows during pregnancy significantly increased the contents of Mg (26.81%, *p* = 0.03), Zn (26.67%, *p* = 0.05), and Cu (18.37%, *p* = 0.035) in the placentas (Figure 3B). Moreover, dietary supplementation with FS in the late gestation period of the sows increased the contents of Fe (79.14%, *p* = 0.012), Cu (329.41%, *p* < 0.001), and Mn (396.61, *p* < 0.001) in the umbilical cord serum (Figure 3C).

### 3.7. Maternal Ferrous Sucrose Supplementation Improved Circulating Iron and Hepatic Iron Stores of Neonatal Piglets

**Figure 4:**
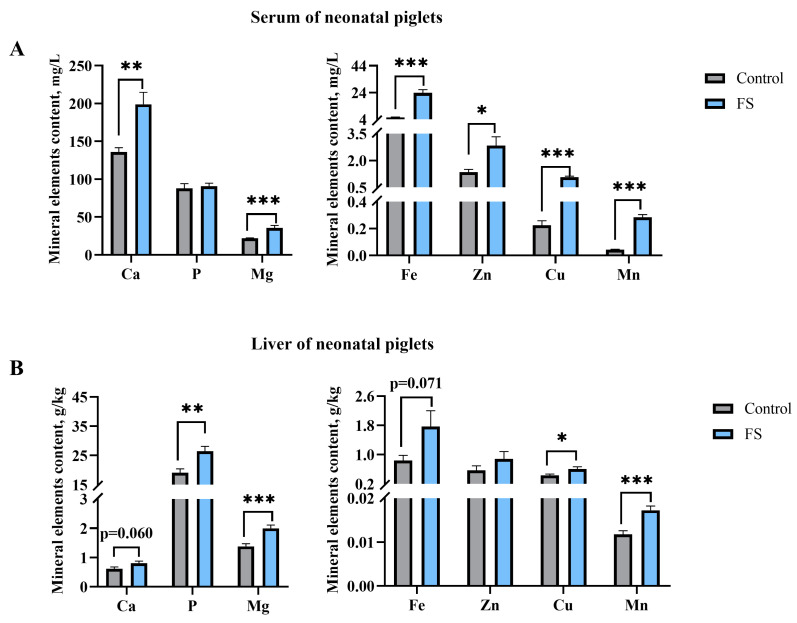
Effects of supplementation of pregnant sows with ferrous sucrose on the mineral element content in the serum and liver of neonatal piglets: (**A**) mineral element content in the serum of neonatal piglets; (**B**) mineral element content in the liver of neonatal piglets. FS = ferrous sucrose, Ca = calcium, P = phosphorus, Mg = magnesium, Fe = iron, Zn = zinc, Cu = copper, Mn = manganese. Data are presented as mean with SEM. Statistical significances were set at * *p* ≤ 0.05, ** *p* ≤ 0.01, and *** *p* ≤ 0.001 by the independent *t*-test.

As shown in Figure 4, the supplementation of the sows with FS in late gestation significantly increased the serum contents of Ca (46.38%, *p* = 0.002), Mg (63.16%, *p* = 0.001), Fe (331.69%, *p* < 0.001), Zn (109.63%, *p* = 0.013), Cu (386.36%, *p* < 0.001), and Mn (435.85%, *p* < 0.001) in the jugular vein of the neonatal piglets (Figure 4A). The results of the mineral element content in the liver of the neonatal piglets are similar to those in the serum. The results indicated that maternal supplementation with FS in late gestation significantly increased the contents of P (38.41%, *p* = 0.004), Mg (44.93%, *p* = 0.001), Cu (41.86%, *p* = 0.038), and Mn (46.35%, *p* = 0.001) in the liver of the neonatal piglets (Figure 4B).

### 3.8. Maternal Ferrous Sucrose Supplementation Inhibited mRNA Expression of Transferrin Receptor 1 and Hepcidin in the Placenta

**Figure 5:**
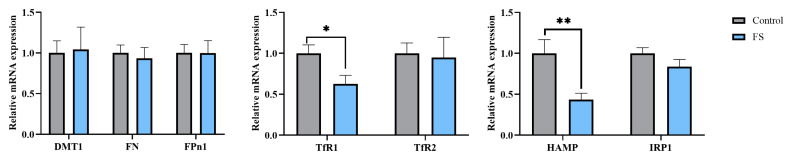
The effect of supplementation of pregnant sows with ferrous sucrose on placental iron-transporter-related gene expression. FS = ferrous sucrose, DMT1 = divalent metal transporter 1, FN = ferritin, FPn1 = ferroportin1, TfR1 = transferrin receptor 1, TfR2 = transferrin receptor 2, HAMP = hepcidin, IRP1 = iron regulatory protein 1. Data are presented as mean with SEM. Statistical significances were set at * *p* ≤ 0.05, ** *p* ≤ 0.01 by the independent *t*-test.

As shown in Figure 5, we found that supplementation with FS of the sows in the last third of gestation significantly down-regulated the relative mRNA expression of placental hepcidin and transferrin receptor 1 (TfR1) (*p* ≤ 0.05).

## 4. Discussion

Plant polysaccharides and oligosaccharides have attracted much attention due to their solubility, bioactivities, and easy absorption in the intestine [34] and are used as chelating ligands of iron for iron deficiency research in animals and humans [28,30,35,36]. Previous studies have shown that polysaccharide iron complexes are becoming a novel comprehensive nutritional supplement to address nutrition and iron requirements [37,38]. In our recent research, it was found that supplementing sows with 140 mg/kg of an enteromorpha polysaccharide-Fe (III) complex (EP-Fe) from late gestation to the end of lactation can improve placental iron transport and the iron content in colostrum to enhance the growth performance and iron storage capacity of newborn piglets [28]. At the same time, using 3-day-old early weaned piglets as a model of iron deficiency anemia, Feng et al. found that adding EP-Fe (10 mg Fe d^−1^) to artificial milk can effectively supplement iron and promote intestinal development and improve growth performance in piglets [30]. We found that supplementing sows with FS from 95 days of pregnancy to delivery improved the reproduction performance of sows and promoted the iron stores of the neonatal piglets in this study, which is basically consistent with the research on EP-Fe mentioned above. This may be related to the characteristics of carbohydrates. Natural polysaccharides have good hydrophilicity and biocompatibility, and their functions can be enhanced through metalization or structural chemical modification [39]. Sucrose is a metal ion chelating agent with excellent stability that can increase the absorption of metal ions by contributing hydroxyl electrons to bind with them [24].

SI and liver iron content are the most commonly used indicators for assessing the body’s iron level, and the amount of iron-bound transferrin is known as SI [40]. Previous research has revealed that supplementing iron during pregnancy can increase the SI [18,41] and liver iron stores [17,18,19] of piglets. TS refers to the ratio of SI to transferrin binding capacity (total iron binding capacity). Under physiological conditions, transferrin in plasma has a very high iron binding ability and is not fully saturated with iron (usually TS is about 30%) [42]. In this study, the SI and TS of sows supplemented with FS in late gestation were significantly increased compared to those of sows in the control group. Iron in the blood enters cells as a complex bound to transferrin, which binds to the transferrin receptor (TfR) on the plasma membrane to access cells through endocytosis [43]. Similarly, iron enters the placenta [5]. Accordingly, our results showed that there was an increasing trend in placental iron content in the FS group, and this was the necessary preparation for delivering iron to the fetus. Meanwhile, as expected, compared with the control group, the iron in umbilical cord serum and the SI in the neonatal piglets were also significantly increased, and the hepatic iron stores of neonatal piglets had a strong upward trend in the FS group. The higher SI in fetuses also further laid a good foundation for the growth of fetuses, which was reflected in the significant increase in the litter weight of the neonatal piglets. These findings are consistent with reports from some authors who support the administration of iron treatment in sows during pregnancy to prevent the occurrence of iron deficiency anemia in piglets. Li et al. found that sows supplemented with 110 mg/kg ferrous glycine chelate from 85 days of pregnancy to delivery showed better Hb concentration, tissue iron content, and blood biochemical indicators in their newborn piglets compared to other forms of supplementation [18]. Buffler et al. confirmed that supplementing 142 mg/kg ferrous sulfate in the basal diet of sows from fertilization to delivery significantly improved the litter size and weight of neonatal piglets [19]. The increase in serum TS in sows indicates accelerated iron mobilization, which is also aimed at efficiently transferring iron from the mother to the offspring to meet fetal growth.

The placenta, the interface between a mother and fetus, mediates nutrient transport from mother to fetus, including the transport of iron [44]. The unidirectional transfer of iron from the mother to the fetal side through the placenta is mainly controlled by TfR, divalent metal transporter 1 (DMT1), and ferroportin (Fpn), which are located at the top and basal lateral membranes of the syncytial trophoblast, respectively [45]. This mode of iron transfer is also regulated by hepcidin [46]. Generally, hepcidin is the iron regulatory hormone secreted by hepatocytes and controls plasma iron concentration by regulating the activity of the iron exporting protein Fpn in cells, such as intestinal epithelial cells, hepatocytes, etc. [47]. There is a classic feedback regulatory loop between iron and hepcidin. When iron is accumulated to a certain extent, hepatocytes generate more hepcidin, thereby inhibiting further absorption of iron from intestinal epithelial cells and the release of iron from storage. When the body lacks iron, less hepcidin is produced by hepatocytes, stimulating more iron to access the blood [47]. However, according to research reports, hepcidin is also expressed in many other mammalian cells, including the placenta, in addition to hepatocytes [46]. In this study, we noticed that during the efficient transfer of iron from the sows to their fetuses (such as elevated SI levels in umbilical cord and piglets, as well as an increasing trend in liver iron stores in piglets), the placental hepcidin in the FS group was significantly reduced. In fact, according to reports, not only mothers with anemia [48] but also mothers with sufficient iron stores [49] exhibit a low expression of hepcidin during childbirth [50]. Due to the special physiological period of pregnancy, in the middle and late stages of pregnancy, severe inhibition of hepcidin is aimed at increasing maternal iron mobilization to promote fetal development, while expanding maternal red blood cell count [51]. However, in this study, the SI of sows supplemented with FS significantly increased, but hemoglobin and hematocrit did not change. The possible explanation for the inhibition of hepcidin is that it is necessary to increase the maternal endogenous iron supply by enhancing iron mobilization from storage, increasing iron absorption from the intestine, and accelerating iron recovery from aging red blood cells to meet the needs of pregnancy.

TfR can mediate iron metabolism. The process of its binding to transferrin loaded with iron through endocytosis transferring iron into cells is strictly controlled by the feedback loop. During this process, hepcidin participates in regulating iron metabolism [42]. Surprisingly, we found that in the placenta of sows supplemented with FS, the mRNA expression of TfR1, an important sensor of extracellular iron, was not increased with an increase in TS of maternal blood and a decrease in placental hepcidin mRNA, but rather significantly decreased. It is speculated that in the special physiological state of pregnancy, in addition to hepcidin, the placenta may have its unique iron transport regulation mechanism, or it may be to protect the fetus from iron overload. In this study, no significant changes were observed in other iron-related transport carriers, which may indicate that FS has unique transport channels. Du et al. found that sucrose calcium chelates enhanced the ability of calcium transport in Caco-2 cells and mouse intestines, which is related to its strong binding to the calcium transport protein PMCA1b located on the cellular basement membrane and its ability to stimulate PMCA1b gene expression [52]. Through in vitro and in vivo studies, Li et al. discovered that zinc sucrose complexes are absorbed by intestinal epithelial cells through active transport, paracellular, and oligosaccharide transport pathways [53]. As some researchers believe, amino acid chelated iron may be transported as a whole through the brush border membrane (BBM) via peptide transporter 1 (PepT1). Their research shows that compared to an equal amount of ferrous sulfate, amino acid chelated iron has a larger and faster intestinal absorption rate and significantly increases PepT1 mRNA levels and protein expression in the intestinal epithelial cells of pigs [54,55]. This suggests that ferrous sucrose may enter cells through oligosaccharide transport pathways.

In the later stages of pregnancy, as iron demand rapidly increases, sows will prioritize iron supply for developing fetuses [56]. Liu et al. found that when sows were supplemented with 140 mg/kg EP-Fe from late gestation to the end of lactation, the serum calcium, phosphorus, and zinc contents were significantly decreased in parturient sows with the increase in liver iron content in newborn piglets [28]. Notably, we found an interesting phenomenon from the results of mineral element contents in sows and placentas or neonatal piglets; that is, the contents of calcium, phosphorus, iron, and zinc in the sows’ serum showed a decreasing trend, and those in the placenta and offspring serum showed an increasing trend, suggesting that the placenta mobilizes calcium, phosphorus, iron, and zinc from the maternal body into fetal circulation. As emphasized by Matte et al. (2020), the placenta provides an active transport and regulatory mechanism for mineral elements to meet the rapid growth of the fetuses [27]. The utilization efficiency of minerals by the fetus is mainly determined by the mother’s supply and their own growth needs [57]. In our study, the increase in iron levels in the blood and liver of neonatal piglets was naturally due to the increased dietary iron supply of sows. Changes in a certain dietary composition can also affect the absorption and metabolism of other nutrients [58]. Usually, the effect of iron on zinc absorption is not significant. However, if high doses of supplemental iron are ingested without food, it is likely that the absorption of zinc is impaired [59]. Iron and zinc are both elements in the first transition series of the periodic table, and they have the same external electronic configuration as manganese. Hill and Matrone [60] proposed a theory that elements with similar physical and chemical properties can exhibit antagonistic effects on each other. Similarly, high iron intake has been shown to have adverse effects on copper status in ruminants [61], guinea pigs [62], and rats [63]. But copper is crucial for the transport of iron in tissues, as it can promote the absorption and transport of iron [64]. The interaction between iron and calcium can reduce the bioavailability of iron [65], while iron supplementation does not affect phosphate or calcium absorption [66]. However, this study showed that the levels of zinc and manganese in the blood of newborn piglets did not decrease due to high iron supply. We speculate that one possible reason is that the increase in iron supply is not sufficient to inhibit the placental transport of zinc and manganese. Secondly, the fetus’s demand for trace element balance can offset some of the antagonistic effects between Fe, zinc, and manganese. Furthermore, the increase in calcium, magnesium, zinc, copper, and manganese levels in piglet serum may be to maintain rapid fetal growth and the homeostasis of mineral elements in the body.

## 5. Conclusions

Supplementing pregnant sows with 109 mg/kg ferrous sucrose in the diet does not alter maternal red blood cell generation but can increase maternal serum iron and transferrin saturation. At the same time, it can promote weight gain and hepatic iron stores in neonatal piglets.

## Figures and Tables

**Table 1:** Composition and nutrient levels of diets (air-dry basis).

Item	Quantity
Ingredients, %	
Yellow corn	65.025
Soybean meal	11.20
Soybean hull	6.00
Steam fish meal	5.00
Soybean oil	1.00
Expanded flaxseed	2.00
Fine stone powder	0.80
Expanded soybean	5.00
Sucrose	1.00
L-threonine	0.12
DL-methionine	0.06
Tryptophan	0.05
L-lysine HCl	0.225
NaCl	0.36
NaHCO_3_	0.18
CaHPO_4_	0.98
Premix ^1^	1.00
Total	100
Nutrient composition ^2^, %	
NE ^3^, MJ/kg	12.58
Crude protein	16.58
Crude fiber	4.5
Total calcium	0.898
Total phosphorus	0.641
Lysine	1.04
Threonine	0.72
Methionine	0.22
Valine	1.05
Isoleucine	0.72
Leucine	1.46
STTD phosphorus ^3^	0.38
Iron, mg/kg	258.09

^1^ The premix provided the following per kg of diets: 4000 IU of vitamin A, 800 IU of vitamin D_3_, 44 IU of vitamin E, 0.2 mg of biotin, 1250 mg of choline, 1.3 mg of folic acid, 10 mg of niacin, 12 mg of pantothenic acid, 3.75 mg of riboflavin, 1.0 mg of thiamine, 15 mg of vitamin B6, 21 µg of vitamin B12, 14 mg of Cu as CuSO_4_·5H_2_O, 120 mg of Zn as ZnSO_4_·H_2_O, 40 mg of Mn as MnSO_4_·H_2_O, 0.2 mg of I as KI, 0.2 mg of Se as Na_2_SeO_3_, 129.3 mg of Fe as FeSO_4_·7H_2_O. ^2^ The nutrient composition was analyzed values. ^3^ Net energy and STTD phosphorus in the diet were calculated values.

**Table 2:** Sequence of primers used in this study.

Gene ^1^	Nucleotide Sequence of Primers (5′–3′)	Size (bp)
FN	F: CGGGACAGAAGAGAATCCCC R: GTCCAAGAACCGGCGAAGTA	166
Fpn1	F: TACCAACGGGGTACTTTGCC R: AGTGGGGAATGCAATTCAGGA	217
TfR1	F: GGCTGTATTCTGCTCGTGGA R: AGCCAGAGCCCCAGAAGATA	195
DMT1	F: GCAGGTGGTTGACGTCTGTA R: CACGCCCCCTTTGTAGATGT	100
TfR2	F: GTGATGGAGACCCCCTTGTG R: GCCCATTATGAAAGGCGCTG	161
HAMP	F: ATCCCAGACAAGACAGCTCAC R: CCCACAGATTGCTTTGCGAC	151
IRP1	F: GCGGCTCTTGACCAGATACA R: AGGGTCGTGCCTTCCTCTAT	201
β-actin	F: CTGCGGCATCCACGAAACT R: AGGGCCGTGATCTCCTTCTG	132

^1^ FN = ferritin; Fpn1 = ferroportin1; TfR1 = transferrin receptor 1; DMT1 = divalent metal transporter 1; TfR2 = transferrin receptor 2; HAMP = hepcidin; IRP1 = iron regulatory protein 1.

**Table 3:** The blood hemoglobin and hematocrit of sows before and after the study.

Period	Blood Parameters	Control	FS	*p*-Value
Before the study (day 95 of gestation)	Hemoglobin, g/L	100.28 ± 3.44	103.47 ± 1.77	0.372
	Hematocrit, %	29.50 ± 1.02	30.40 ± 0.53	0.397
After the study (parturition)	Hemoglobin, g/L	108.45 ± 3.71	101.56 ± 1.52	0.112
	Hematocrit, %	31.50 ± 1.28	29.81 ± 0.40	0.236

FS = ferrous sucrose. Data are presented as mean ± SEM. Statistical significances were set at *p* ≤ 0.05 by the independent samples *t*-test.

**Table 4:** Effects of maternal FS supplementation on reproductive performance of sows.

Reproductive Performance	Control	FS	*p*-Value
Litter size, n	13.56 ± 0.48	14.93 ± 0.73	0.113
Number born alive, n	13.28 ± 0.44	13.64 ± 0.58	0.613
Number of stillbirths, n	0.28 ± 0.11	0.71 ± 0.34	0.238
IUGR ^1^, n	0.83 ± 0.29	0.64 ± 0.20	0.618
Birth litter weight, kg	16.54 ± 0.52	19.97 ± 0.95	0.002
Average weight, kg	1.24 ± 0.04	1.36 ± 0.05	0.078

^1^ IUGR (intrauterine growth restriction) piglets refer to piglets whose birth weight is 1.5 SD lower than the average litter weight. Litter size = number born alive + number of stillbirths + number of mummies; IUGR = number born alive - number of healthy piglets; birth litter weight = weight of born alive + weight of stillbirths + weight of mummies; average weight = birth litter weight/litter size. FS = ferrous sucrose. Data are presented as mean ± SEM. Statistical significances were set at *p* ≤ 0.05 by the independent samples *t*-test.

## Data Availability

Data are available on reasonable request from the authors.
